# RSV attenuates epithelial cell restitution by inhibiting actin cytoskeleton-dependent cell migration

**DOI:** 10.1152/ajplung.00118.2021

**Published:** 2021-05-19

**Authors:** Debra T. Linfield, Nannan Gao, Andjela Raduka, Terri J. Harford, Giovanni Piedimonte, Fariba Rezaee

**Affiliations:** ^1^Cleveland Clinic Lerner College of Medicine of Case Western Reserve University, Cleveland, Ohio; ^2^Department of Inflammation and Immunity, Lerner Research Institute, Cleveland Clinic Foundation, Cleveland, Ohio; ^3^Department of Biochemistry and Molecular Biology, Tulane University School of Medicine, New Orleans, Louisiana; ^4^Center for Pediatric Pulmonary Medicine, Cleveland Clinic Children’s, Cleveland, Ohio

**Keywords:** epithelial restitution, focal adhesion, respiratory syncytial virus, Rho-associated kinase, wound healing

## Abstract

The airway epithelium’s ability to repair itself after injury, known as epithelial restitution, is an essential mechanism enabling the respiratory tract’s normal functions. Respiratory syncytial virus (RSV) is the leading cause of lower respiratory tract infections worldwide. We sought to determine whether RSV delays the airway epithelium wound repair process both in vitro and in vivo. We found that RSV infection attenuated epithelial cell migration, a step in wound repair, promoted stress fiber formation, and mediated assembly of large focal adhesions. Inhibition of Rho-associated kinase, a master regulator of actin function, reversed these effects. There was increased RhoA and phospho-myosin light chain 2 following RSV infection. In vivo, mice were intraperitoneally inoculated with naphthalene to induce lung injury, followed by RSV infection. RSV infection delayed reepithelialization. There were increased concentrations of phospho-myosin light chain 2 in *day 7* naphthalene + RSV animals, which normalized by *day 14*. This study suggests a key mechanism by which RSV infection delays wound healing.

## INTRODUCTION

The airway epithelium serves as the first line of defense against inhaled allergens, particles, and viruses and therefore is under constant threat of injury by environmental exposure (1). Airway epithelial injury is manifested as a focal loss of cells and denuded areas and is known to occur as a consequence of inhalation of pathogens, airborne particulate matter, cigarette smoke, and allergens (2–6). Injury might also occur in pediatric intensive care units (PICU) during mechanical ventilation as a result of barotrauma or volutrauma (7–10). These wounds may allow for greater penetration of inhaled allergens and particles into the subepithelial space (11) or as an opening for viral or bacterial infections (12). As such, epithelial wound repair and regeneration after injury, known as epithelial restitution, is crucial for restoring barrier integrity and maintenance of normal function of the respiratory tract (13, 14).

Epithelial restitution is a multistep process requiring robust cross talk between major cellular functions. It involves several essential cellular processes including spreading and migration of cells into the wounded area, followed by proliferation and differentiation of epithelial cells (15). Migration of epithelial cells from the wound edges represents a key early restitution event, allowing for quick covering of denuded airway areas (13, 14). Dysregulated wound repair capacity and loss of barrier integrity have been observed in patients with pulmonary disorders including asthma (16, 17), chronic obstructive pulmonary disorder (2, 18–20), and cystic fibrosis (21–23). Furthermore, viral infections such as human rhinoviruses and influenza A virus have been shown to decrease self-repair processes of airway epithelial cells (24, 25).

Respiratory syncytial virus (RSV) is the most common cause of acute lower respiratory tract infection in children worldwide, infecting nearly all children by their second birthday (26). RSV mainly infects airway epithelial cells, causing profound inflammation and marked changes in epithelial cell physiology. Clinical studies have shown that RSV can infect airway epithelium injured by various environmental insults. For instance, multiple studies have described RSV outbreaks in neonatal intensive care units and PICU due to barotrauma and volutrauma (27, 28). Epithelial damage, further exacerbated by RSV infection, may be one mode allowing for bacterial superinfection (12) or greater penetration of inhaled allergens into the subepithelial space (11). In fact, observational studies have found that 36%−44% of children admitted to PICU with severe RSV infection harbor bacterial pathogens in their lower airways (29–32), perhaps implying that one mechanism of bacterial superinfection is RSV infection enabling easier penetration for other pathogens and stimuli.

Our previous studies revealed that RSV disrupts epithelial cell contacts, known as apical junctional complexes (AJC) (33–35). RSV infection markedly induces remodeling of the actin cytoskeleton (33). The actin cytoskeleton exists as a contiguous band of filaments connected to epithelial AJC and is responsible for maintaining cell stability and shape and regulating cell migration (36, 37). Epithelial cell actin homeostasis is regulated in part by actin-binding small Rho GTP-binding proteins (GTPases), which have been extensively studied in cytoskeletal organization and regulation of cell migration (38–40). In particular, one of these Rho GTPases, known as RhoA, activates its downstream effector, Rho-associated kinase (ROCK), at times of mechanical stress (41, 42). ROCK directly phosphorylates myosin light chain (MLC), leading to myosin II activity and actin contraction generation (41). Through these mechanisms, ROCK is responsible for many cell functions, including remodeling of the extracellular matrix (ECM), cell mobility, and actin cytoskeleton organization (43–46). In addition, actin connects to the ECM through large, dynamic protein complexes named focal adhesions (FAs), which play a major role during cell migration (47, 48).

The impact of RSV on the restoration processes of an injured airway is unknown. In the current study, we postulated that RSV infection attenuates restoration of the airway epithelia via mechanisms affecting actin cytoskeleton organization and Rho GTPase signaling. We examined the effects of RSV infection on airway epithelial restitution, and in particular its early phases involving cell migration. We analyzed the role of downstream molecular mechanisms by which RhoA signaling induces RSV-mediated hypomotility. In addition, we investigated the effects of RSV infection on airway epithelial restitution in vivo.

## MATERIALS AND METHODS

### Antibodies and Other Reagents

The following primary monoclonal and polyclonal antibodies were used to detect cytoskeletal and FA proteins by immunofluorescent labeling and immunoblot analysis: anti-GAPDH monoclonal antibody (Cat. No. ab8245, Abcam, Cambridge, MA), anti-secretoglobin family 1A member 1 (CC10/scgb1a1; Cat. No. 213202, Abcam), anti-E-cadherin monoclonal antibody (Cat. No. 610181, BD Bioscience, San Jose, CA), total paxillin (PAX; Cat. No. 2542, Cell Signaling, Danvers, MA), phospho-PAX Tyr^118^ (pPAX; Cat. No. 2541, Cell Signaling), total focal adhesion kinase (FAK; Cat. No. 3285, Cell Signaling), phospho-FAK Tyr^397^ (pFAK, Cat. No. 3283, Cell Signaling), phospho-MLC2 (pMLC2; Cat. No. 3671t, Cell Signaling), MLC2 (Cat. No. 8505s, Cell Signaling), and RhoA (Cat. No. 2117s, Cell Signaling). Alexa Fluor 488 (Cat. No. A12379) and 633 (Cat. No. A22284) phalloidin as well as anti-rabbit and anti-mouse secondary antibodies conjugated to Alexa Fluor 488, 568, or 633 (Cat. Nos. A-21206, A-10042, and A-21050) dyes were obtained from Thermo Fisher Scientific (Waltham, MA). The specificity of antibodies against MLC2 and pMLC2 has been reported previously (49–51). ROCK inhibitor Y-27632 was purchased from Millipore Sigma (Cat. No. SCM075, St. Louis, MO) and resuspended in deionized water. Y-27632 inhibits both ROCK1 and ROCK2 by competing with ATP for binding to the catalytic site (52).

### Cell Culture

16HBE14o (henceforth referred to as 16HBE cells) were provided by Dr. Dieter Gruenert (University of California, San Francisco, CA). This cell line was isolated from a 1-yr-old male heart-lung patient and immortalized with the origin of replication defective simian virus 40 plasmid. Cells were genotyped by Short Tandem Repeat (STR) analysis to verify cell line authentication and were negative for mycoplasma contamination (53). Cells were cultured on flat bottom well plates or grown on collagen-coated Transwell permeable supports (Corning, Tewksbury, MA) under liquid-liquid conditions. Rat tail collagen (type 1) was purchased from BD Biosciences. Primary normal human bronchial epithelial (NHBE) cells isolated from the lungs of a 7-yr-old Hispanic female donor were grown on Transwell membrane inserts under air-liquid interface conditions as previously described (33, 34, 54). All cell lines were authenticated before experiment initiation.

### RSV Infection

RSV derived from RSV A2, which expresses red fluorescent protein upon replication (rrRSV), was a kind gift from Dr. Mark Peeples (Nationwide Children’s Hospital Research Institute, Columbus, OH) and Dr. Peter Collins (National Institute of Health, Bethesda, MD) (55, 56). Virus inactivation was performed by exposure to UV-B radiation for 20 min. This abolishes the virus’s ability to replicate, which was confirmed by a plaque-forming assay (33, 35).

### Immunofluorescent Labeling and Confocal Microscopy

For immunofluorescent staining, cells were fixed in 4% paraformaldehyde and subjected to immunofluorescence labeling as previously described (33–35). Immunolabeled cell monolayers were examined using a Leica TCS-SP spectral laser scanning confocal microscope.

### Wound Healing Assays

Assays were performed as previously described (18, 57–59). Once confluent, cells were treated with medium control, UV-inactivated RSV (UV-RSV), or live rrRSV [multiplicity of infection (MOI): 0.1−1] for 48 h. Select wells were incubated with 10 μM ROCK inhibitor Y-27632 for 1 h before wounding. Mechanical “scratch” injury was induced by scraping the cell layer with a sterile pipette tip, creating a wound with a diameter of roughly 700−750 µm (60, 61). After wounding, cell debris was removed by further rinsing the monolayers with media. Photographs were taken at the same location along the wound immediately after creation and multiple intervals until closure. Wound surface area was measured using ImageJ imaging software (62) to assess closure rate of the denuded space.

### Immunoblot and Western Blot Analyses

Confluent 16HBE cells were exposed to control medium, UV-RSV, or RSV, and 48 h after infection, mechanical “scratch” injury was induced by scraping the cell layer with a sterile pipette tip as described in *Wound Healing Assays*. Cell lysates were collected with RIPA lysis buffer (with Halt protease and phosphatase inhibitors, Thermo Scientific, Waltham, MA) at 10 min and 2 h postwound along with the control group. Western blot analysis was performed as previously described (35, 54). Briefly, total protein concentration was determined by a Pierce bicinchoninic acid (BCA) Protein Assay kit (Thermo Scientific), separated by SDS-PAGE, and transferred to polyvinylidene difluoride (PVDF) membranes (Bio-Rad Laboratories, Hercules, CA). Membranes were then incubated with the indicated primary antibodies overnight at 4°C and with horseradish peroxidase-conjugated secondary antibodies for 1 h at room temperature. Blots were visualized with regular or enhanced chemiluminescence (Thermo Scientific), and immunoactive bands were imaged using MyECL imager (Thermo Scientific). The pixel density of each band was estimated with Image Studio Lite software (LI-COR Biosciences, Lincoln, NE) and normalized to either total MLC2 or the lane loading control, GAPDH. Results were expressed as a ratio of pMLC2 to MLC2 and MLC2 to GAPDH, respectively.

An uncropped Western blot figure for each antibody showing the entire lane with molecular weight markers is provided as Supplemental Fig. S1 (see https://doi.org/10.6084/m9.figshare.14579871.v1).

### Attachment and Spreading Assays

Adherent 16HBE cells were removed from the culture substrate by treatment with TrpLE Express recombinant cell dissociation enzymes (Gibco-Thermo, Waltham, MA). For the attachment assay, which uses detection and counting of bound cells (63), cells were resuspended in whole cell medium in the presence or absence of 10 μM ROCK inhibitor Y-27632, as per the manufacturer’s guidelines. Equal numbers of cells were then replated on collagen type I-coated 24-well tissue culture plates (CytoOne) and incubated at 37°C for 2 h. Plates were washed with PBS and fixed with room temperature paraformaldehyde. Cells were identified with DAPI fluorescent nuclear marker (VectaShield). Images were taken using a fluorescence microscope and quantitatively assessed for the presence of cells that had adhered to the well bottom with ImageJ imaging software (62). A spreading assay (64) was used to measure the flattening of adherent cells. After 48 h of RSV infection, cells were resuspended in whole cell medium in the presence or absence of 10 μM ROCK inhibitor. Equal numbers of cells were replated on collagen-coated Transwell membrane inserts (Corning) or flat-bottom plates and incubated at 37°C for 2 h. Following this, wells were washed with PBS and fixed with room temperature paraformaldehyde. Membrane filters were excised and fluorescently labeled with filamentous actin (F-actin) probe, phalloidin-488, or phalloidin-633 (Thermo) and photographed under phase-contrast bright-field conditions to assess morphology. The cell surface area was quantified with ImageJ software and normalized to the control.

### Proliferation Assay

Cell proliferation was assessed by two methods: using a commercially available 5-ethynyl-2′-deoxyuridine (EdU) kit (Abcam) and counting. For the EdU assay, cells at 60%−70% confluency were fixed, permeabilized, and incubated with EdU, a thymidine analog that becomes incorporated into newly synthesized DNA according to the manufacturer’s protocol. EdU was covalently cross-linked with fluorescent azide, iFluor 488. DNA was stained with DAPI, and EdU fluorescence was read at an excitation/emission wavelength of 491/520 nm. Photographs were taken under fluorescent light and used to quantify the ratio of EdU-positive cells to total cells. For counting, after 48 h of exposure of epithelial cells to control medium, UV-RSV, or RSV, cells were gently detached with TrypLE Express, stained with trypan blue, and counted by a hemocytometer.

### Naphthalene Murine Lung Injury Model

Lung injury was induced in 6- to 8-wk-old female C57BL/6 mice by a single intraperitoneal injection of 200 mg/kg naphthalene (Sigma) dissolved in corn oil (65–67). Studies by Chen et al. (66–68) have demonstrated that this model selectively ablates club cells of the proximal and distal conducting airways, resulting in the formation of epithelial wounds. In a series of pilot experiments to determine the precise time course of airway epithelial injury and repair, mice were euthanized at the indicated days postinjection, and their lungs were harvested for histological and immunolabeling analyses. In subsequent experiments, animals were administered either naphthalene or vehicle and, at *day 2* postinjection, received an intranasal inoculation of 9.6 × 10^6^ plaque-forming units of RSV as described in our recent publication (35). Mice were harvested on *day 7* (corresponding with a “healing” epithelium) and *day 14* (corresponding with a “healed” epithelium). Lung tissue and bronchoalveolar lavage fluid were collected and used to evaluate lung inflammation by quantification of infiltrated leukocytes and protein translocation into the lung. Airway epithelial injury and restitution were evaluated by assessing hematoxylin and eosin-stained paraffin-embedded lung sections and by antigen retrieval and immunolabeling of club cells with a specific marker, CC10/scgb1a1 (69), the pan-epithelial marker E-cadherin, and pMLC2.

### Ethics Statement

Human primary epithelial cells were isolated from human tissue from deceased pediatric donors. Tissue was provided by the International Institute for the Advancement of Medicine according to procedures approved by the Cleveland Clinic. As such, the human tissue is exempted from requiring Institutional Review Board approval as the use of this tissue is not considered a human study by the Cleveland Clinic Foundation. All animal procedures used in this study adhered to the National Institutes of Health Guide for the Care and Use of Laboratory Animals and were reviewed and approved by the Institutional Animal Care and Use Committee (approved protocol 2018-2030) of the Lerner Research Institute at the Cleveland Clinic Foundation. This facility is accredited by the Association for the Assessment and Accreditation of Laboratory Animal Care (Accreditation No. 000383) and is in compliance with federal law and National Institutes of Health regulations.

### Data Analyses

Data were analyzed using Prism software (GraphPad, San Diego, CA) and Microsoft Excel. Data are representative of three or more experiments and are presented as means and SD. For comparison of two groups with parametric data, Student’s two-tailed *t* test or paired Student’s one-tailed *t* test were used. For comparison of multiple groups, we performed one-way ANOVA followed by Dunnett’s post hoc test for all groups of the experiment.

## RESULTS

### RSV Attenuates Epithelial Cell Migration after Wounding

**Figure 1.**
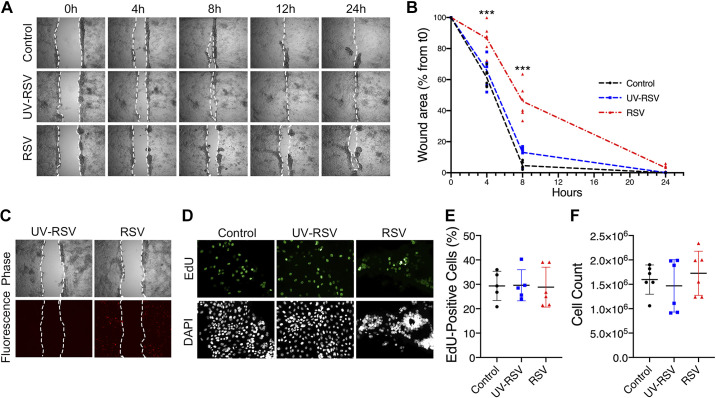
Respiratory syncytial virus (RSV) infection delays airway epithelial wound healing in vitro. 16HBE14o (16HBE) cells were grown to confluence and infected with either live RSV [0.5 multiplicity of infection (MOI)], UV-inactivated RSV (UV-RSV), or medium control. After 48 h of viral infection, cell monolayers were mechanically wounded. Migration into the wounded monolayer was examined at the indicated times by phase-contrast microscopy. Images of representative wounds (*A*) and the calculated rate of wound closure over time (*B*) are shown. *C*: representative images of rrRSV-infected cells expressing red fluorescent protein, as evidence of active viral infection. 16HBE cells were seeded to semiconfluence and infected with either live RSV (0.5 MOI), UV-RSV, or medium control. At 48-h postinfection, cells were labeled with 5-ethynyl-2′-deoxyuridine (EdU), and numbers of EdU-positive cells were visualized and counted (*D* and *E*). 16HBE cells were grown to confluence and inoculated with control medium, UV-RSV, or rrRSV (0.5 MOI). After 48 h, cells were gently detached with TrypLE Express, stained with trypan blue, and counted by a hemocytometer (*F*). Data are presented as all data points in *A* and as means ± SD for *E* and *F*; *n *=* *3 independent experiments. ANOVA was conducted. ***P < 0.001 compared with control. t0, *time 0*.

The present study was designed to investigate whether infection with RSV attenuates the repair process of airway epithelial cells. In initial experiments, we examined the effect of RSV on wound healing in vitro by inoculating confluent 16HBE cells grown on six-well plate with control medium, UV-RSV, or rrRSV at a MOI of 0.5 for 48 h, followed by mechanical scratching of the cell monolayers as described in materials and methods. This MOI was chosen based on our previous studies and pilot experiments showing the lowest concentration of RSV infection with a significant impact on wound healing (33–35, 54). The repair rate was assessed by serial imaging, measuring the closure of the wound over 24–26 h and comparing it with the *time 0* of each wound. Mechanical scratch wounds of 16HBE cells infected with RSV were significantly slower to close as cells took longer to migrate to the wound than those inoculated with control medium. Cells exposed to UV-RSV displayed a similar rate of wound closure to the medium control group (Fig. 1, *A* and *B*), which is evidence that live replicating virus is required to delay the wound healing. Likewise, Fig. 1*C* shows a representative image of rrRSV-infected cells, expressing red fluorescent protein. The localization of rrRSV-infected cells was not affected by the scratch wound, and the cells were dispersed throughout the culture.

In addition to migration, cell death or proliferation might contribute to the epithelial wound repair rate. RSV infection has not been shown to cause cell death in multiple studies (33, 70). To evaluate the effect of RSV infection on cell proliferation, we used EdU, a thymidine analog that becomes incorporated into newly synthesized DNA. 16HBE cells were seeded on a permeable Transwell membrane. After 24 h, semiconfluent cells were inoculated with control medium, UV-RSV, or rrRSV. At 48 h postinfection, cells were labeled with EdU according to the manufacturer’s protocol, and EdU-positive cells were enumerated. Visualization under fluorescence microscopy and EdU-positive cell count revealed similar numbers between all groups (Fig. 1, *D* and *E*). In parallel, 16HBE cells were seeded on permeable membrane and inoculated with control medium, UV-RSV, or rrRSV. At 48 h postinfection, cells were detached and counted. The counts were comparable among all groups (Fig. 1*F*). This suggests that cell proliferation and death do not play a role in the wound repair rate.

### RSV Attenuates Primary Human Epithelial Cell Wound Healing

**Figure 2.**
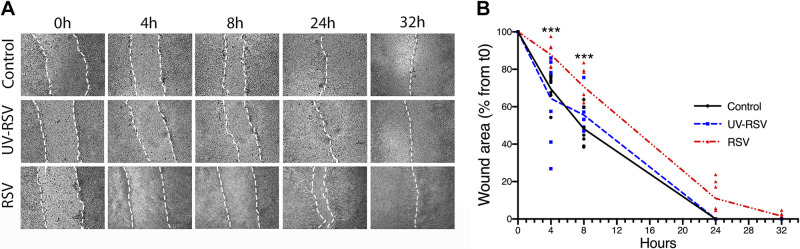
Respiratory syncytial virus (RSV) infection delays airway epithelial wound healing of primary human epithelial cells grown under air-liquid interface (ALI) conditions. Primary normal human bronchial epithelial cells were grown on permeable membranes, differentiated under an air-liquid interface, and infected with either live RSV (0.5 multiplicity of infection), UV-inactivated RSV (UV-RSV), or medium control. After 48 h of viral infection, cell monolayers were mechanically wounded, and migration into the wounded monolayer was examined at the indicated times by phase-contrast microscopy. Images of representative wounds (*A*) and the calculated rate of wound closure over time (*B*) are shown. Data are presented as all data points; *n *=* *3 independent experiments. ANOVA was conducted. ***P < 0.001 compared with control. t0, *time 0*.

To study wound repair in primary cells, NHBE cells were grown on Transwells and maintained in an air-liquid interface until differentiated, followed by RSV infection and mechanical wounds. Similar to 16HBE cells, RSV infection of primary epithelial cells slowed the wound closure compared with the control and UV-RSV groups (Fig. 2, *A* and *B*). The rate of wound closure in primary cells for both noninfected and infected groups was slower compared with 16HBE cells. Wounds of all RSV-infected 16HBE monolayers closed by 24–26 h, and wounds of primary cell monolayers closed by 30–32 h. However, when we grew the 16HBE monolayers on Transwells, there were similar wound repair times to NHBE cells (data not shown). Taken together, data showed that 16HBE cells are a suitable and predictive model for investigating wound repair of bronchial airway epithelial cells.

### RSV Increases Cell Attachment and Induces Remodeling of the Actin Cytoskeleton and FA Assembly

**Figure 3.**
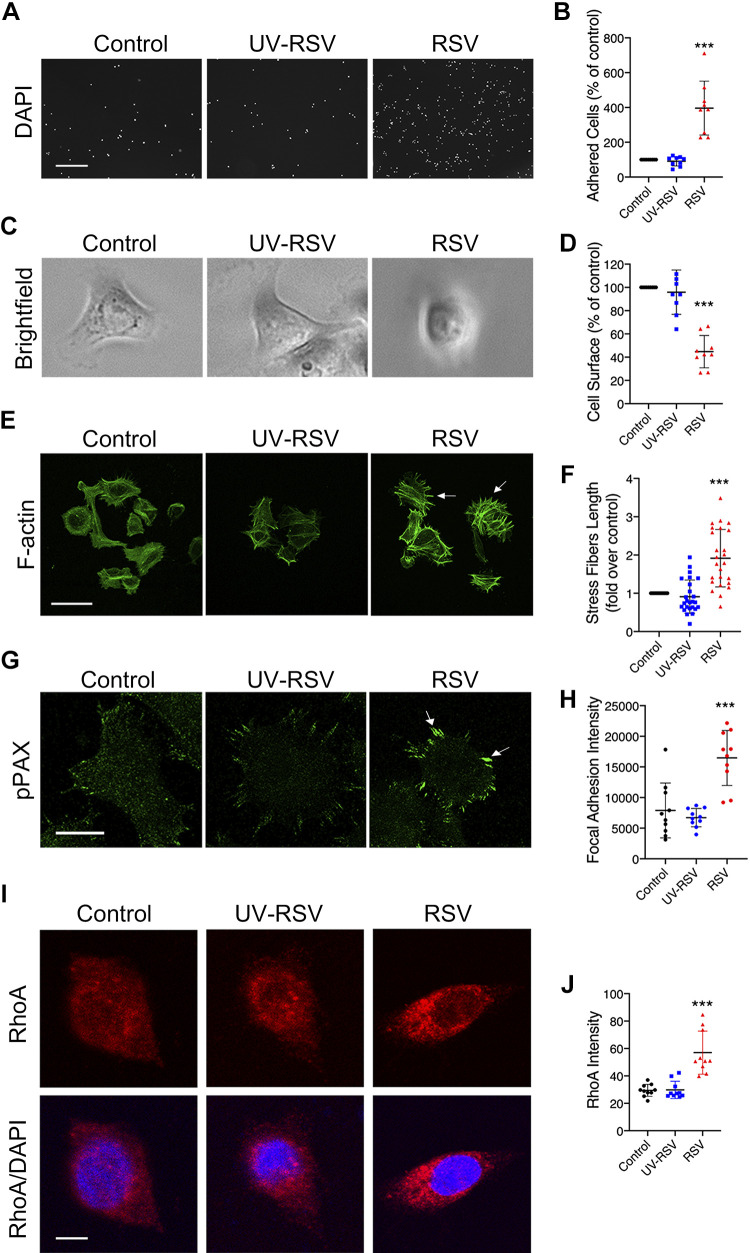
Respiratory syncytial virus (RSV) increases airway epithelial cell adhesion by remodeling of the actin cytoskeleton and through focal adhesion plaque formation. 16HBE14o cells were infected for 48 h with medium control, live RSV (0.5 multiplicity of infection), or UV-inactivated RSV (UV-RSV) followed by cell detachment and seeding on collagen type I-coated plates. After 2 h, nonattached cells were washed off, and attached cells were visualized with DAPI and counted (*A* and *B*). Furthermore, cells were photographed in phase contrast with quantification of cell surface by ImageJ (*C* and *D*). Cells were fixed and probed for phalloidin, and the length of actin fibers was quantified by ImageJ (*E* and *F*). At the same time, cells were immunolabeled with phospho-paxillin (pPAX; *G*) and RhoA (*I*) antibody, with quantification of pPAX and RhoA intensities using ImageJ (*H* and *J*, respectively). Arrows in *E* point to prominent stress fiber protrusions or the assembly of large focal adhesions in RSV-infected cells. Arrows in *G* show the length of pPAX. Scale bars = 200 μm in *A*, 40 μm in *E*, 10 μm in *G*, and 5 μm in *I*. Data are presented as means ± SD; *n *=* *3 independent experiments. Student’s two-tailed *t* test analysis and ANOVA were conducted. ***P < 0.001 compared with control.

Epithelial cells attach to each other and the ECM. During the cell migration phase of wound healing, the epithelium interacts with the ECM to rapidly cover the wound, a process that is independent of cell mitosis (71, 72). However, excessive interlinkage with the ECM can prohibit cell migration. To explain the mechanisms underlying the observed slower migration in RSV-infected epithelium, we sought to determine the effects of RSV infection on epithelial cell adherence to the ECM. Cell adhesion assays were performed as described in materials and methods. Briefly, control and RSV-infected 16HBE cells were removed from the culture substrate at 48 h, resuspended in whole cell medium, and replated on collagen type I-coated plates. After 2 h, plates were washed, fixed, and stained with DAPI. We found that RSV infection increased the number of epithelial cells attached to collagen type I, a major protein component of the ECM, compared with medium control and UV-RSV (Fig. 3, *A* and *B*). Since we are using rrRSV, which expresses red fluorescent protein upon replication, we could identify the infected cells. When we examined the wells containing RSV under fluorescence microscopy, we saw a mixture of cells both with and without red fluorescent protein (RFP) tag expression (data not shown). This indicates that increased attachment of cells to the ECM is more than from a direct cytopathic effect. Previous studies have shown that the cells at the wound edge become elongated and stretched to cover the wounded area (71). Phase-contrast microscopy also revealed that uninfected or UV-RSV-inoculated cells adopted a flattened morphology with cell protrusions, whereas those infected with RSV did not spread upon adherence to the ECM (Fig. 3, *C* and *D*). To gain a further understanding of changes in the cytoskeleton, the organization of the actin cytoskeleton was examined in cells by fluorescent labeling of F-actin with phalloidin. This showed assembly of robust F-actin stress fibers on the cell base in RSV-infected cells quantified by ImageJ (Fig. 3, *E* and *F*). pPAX is an adaptor protein in FAs. There was an increase shown on confocal microscopy in pPAX suggesting that hyperadhesiveness was mediated by the assembly of large FAs and stress fibers (Fig. 3*G*). Confocal microscopy indicated that FAs were elongated (Fig. 3*G*, arrows), which were quantified by ImageJ (Fig. 3*H*). PAX coordinates the activation of RhoA, and, therefore, to further confirm these findings, immunofluorescent labeling of RhoA was investigated in control, UV-RSV, and RSV-infected 16HBE monolayers. This showed an increase in RhoA by direct visualization with confocal microscopy in RSV-infected cells compared with the negative control groups (Fig. 3, *I* and *J*).

### RSV Infection Increased pMLC2 Expression of Airway Epithelial Cells

**Figure 4.**
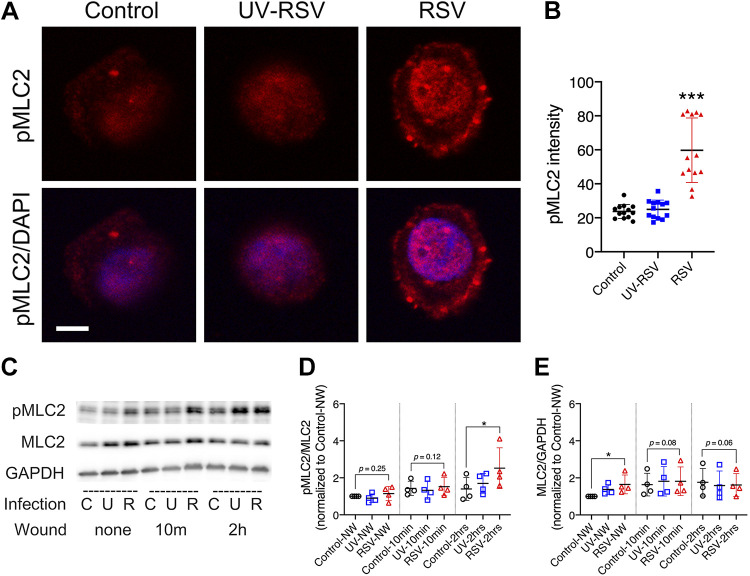
Respiratory syncytial virus (RSV) infection promotes phospho-myosin light chain 2 (pMLC2) expression. 16HBE14o (16HBE) cells were infected for 48 h with medium control (C), live RSV (R; 0.5 multiplicity of infection), or UV-inactivated RSV (UV-RSV; U) followed by cell detachment and seeding on collagen type I-coated plates. After 2 h, nonattached cells were washed off, and attached cells were fixed and immunolabeled with pMLC2 antibody (*A*). Scale bar = 5 μm. Total cell fluorescence was measured using ImageJ software (*B*). Western blot analysis of 16HBE control and RSV-infected cells was performed using total cell lysates. Expression of pMLC2 and total MLC2 were analyzed by Western blot, followed by densitometric analysis, normalized to total MLC2 and GAPDH, respectively (*C−E*). Data are presented as means ± SD; *n *=* *3 independent experiments. Student’s two-tailed *t* test was used for the results shown in *B* (*n *=* *4); a paired Student’s one-tailed *t* test was used for the results shown in *D* and *E*. *P < 0.05 and ***P < 0.001 versus the respective control as determined.

Since we saw an increase in stress fiber assembly and RhoA, we sought to investigate the implication of the RhoA-ROCK-pMLC pathway in our model. Control, UV-RSV, and rrRSV cells were collected at 48 h postinfection and replated on chamber wells. Using confocal microscopy and subsequent quantification with ImageJ, we found increased levels of pMLC2 in RSV-infected cells compared with the noninfected and UV-RSV groups (Fig. 4, *A* and *B*). Furthermore, in RSV-infected cells, there was an increase in pMLC2 within the cell membrane and nucleus compared with noninfected control and UV-RSV groups, which had a homogenous pattern on pMLC2 staining. The increased levels and alterations in localization of pMLC2 in RSV-infected cells suggest that this pathway contributes to changes in the actin cytoskeleton and stress fiber and FA assembly after wounding. To further verify the pMLC2 changes in our wound model, we scratched control, UV-RSV, and rrRSV cells after 48 h and performed Western blot analysis and densitometry quantification for pMLC2 and total MLC2 at 0, 10 min, and 2 h after wounding (Fig. 4, *C−E*). Among the nonwounded groups, we observed a slight increase in pMLC2 in the RSV-infected 16HBE group. Wounded cells had an increase in pMLC2 compared with nonwounded groups. Furthermore, RSV-infected cells at 2 h postwounding showed a significant increase in pMLC2 levels compared with the respective control. There were insignificant increases in total MLC2. These data highlight a potential role for the RhoA-ROCK-pMLC pathway in RSV-infected wound healing.

### ROCK Inhibition Attenuates the Effects of RSV on Migration and Adhesion of Airway Epithelial Cells

**Figure 5.**
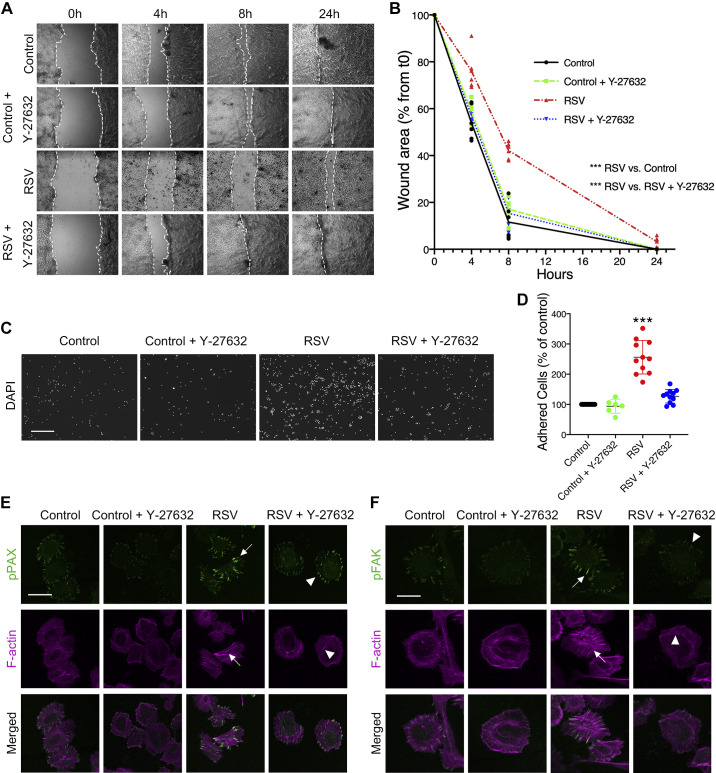
Rho-associated kinase (ROCK) inhibition attenuates the effects of respiratory syncytial virus (RSV) by mitigating the formation of actin stress fibers and focal adhesion bundles. 16HBE14o (16HBE) cells were infected with either live RSV (0.5 multiplicity of infection) or medium control for 48 h. Cells were treated with either vehicle or Y-27632 (10 µM) for 1 h prior to scratching, and wound closure was measured over time. Images of representative wounds (*A*) and the calculated rate of wound closure over time (*B*) are shown. In parallel, cells were detached and reseeded in the presence and absence of Y-27632 (*C* and *D*), and focal adhesion/stress fiber assembly (*E* and *F*) was examined. Arrows in *E* and *F* point to the assembly of large focal adhesions or prominent stress fiber protrusions in RSV-infected cells. Arrowheads point to mitigated formation after exposure to Y-27632. Scale bars = 200 μm in *C* and 60 μm in *E* and *F*. Data are presented as all data points in *B* and as means ± SD in *D*; *n *=* *3 independent experiments. ANOVA was conducted. ***P < 0.001 for all time points aside from 24 h. t0, *time 0*.

Activity of the RhoA-ROCK-pMLC pathway is potently blocked by ROCK inhibitor Y-27632. Pretreatment with 10 µM Y-27632 for 1 h before wound creation almost reversed RSV-dependent inhibition of wound closure (Fig. 5, *A* and *B*). RSV-infected monolayer wounds closed at a slower rate than those exposed to Y-27632. In addition, replating cells in the presence of 10 µM Y-27632 reduced ECM hyperadhesiveness (Fig. 5, *C* and *D*). Finally, stress fiber formation and focal adhesion protrusions of pPAX and pFAK, a nonreceptor tyrosine kinase that helps regulate cell adhesion and migration, were significantly reduced in cells exposed to Y-27632 (Fig. 5, *E* and *F*). Of note, there were negligible differences in the restitution rate or adhesion between control and control + Y-27632 groups.

### RSV Attenuates Epithelial Restitution after Acute Lung Injury In Vivo

**Figure 6.**
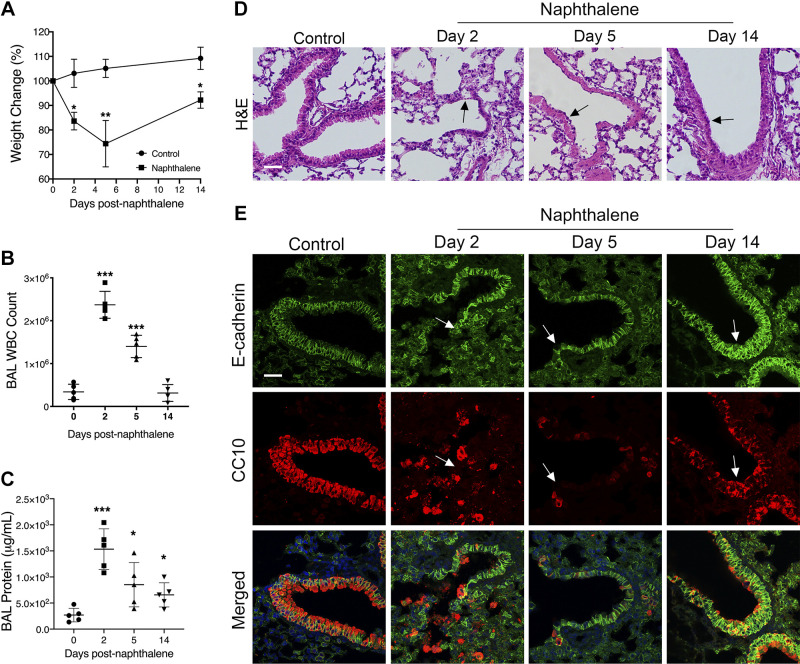
Naphthalene induces acute epithelial damage by ablating club cells and inducing inflammation. Mice were intraperitoneally administered control PBS or naphthalene on *day 0*. On *days 2*, *5*, and *14*, mice were harvested. Mice were weighed daily, and the percentage of body weight change was calculated from body weights at *day 0* (*A*). Transmigrated leukocytes [white blood cells (WBC); *B*] and total protein concentration (*C*) were measured in the bronchoalveolar lavage (BAL). Photomicrographs of hematoxylin and eosin (H&E)-stained lung tissue sections were assessed for histological analysis (*D*). Arrows indicate histopathological changes including peribronchial inflammation and epithelial thickening that recovered by *day 14*. Immunohistochemical analysis of epithelial and club cell markers was conducted (*E*). Arrows in *E* show areas of denuded epithelium that recovered by *day 14*. Scale bar = 40 μm. Data are presented as means ± SD; *n *=* *10 mice per group. ANOVA was conducted. *P < 0.05; **P < 0.01; ***P < 0.001. CC10, secretoglobin family 1A member 1.

To study the impact of RSV infection on the attenuation of epithelial wound healing in a more physiologically relevant model, we used our well-characterized in vivo mouse model of RSV infection (35). Lung injury was induced in 6- to 8-wk-old C57BL/6 mice by a single intraperitoneal injection of 200 mg/kg naphthalene (Sigma) dissolved in corn oil (65, 66, 69). This well-established model selectively ablates club cells of the proximal and distal conducting airways, resulting in the formation of denuded areas of epithelia (40, 68). In a series of pilot experiments performed to determine the precise time course of airway epithelial injury and repair, mice were euthanized at 2, 5, and 14 days after naphthalene injection. Weight change (Fig. 6*A*) and infiltration of white blood cells (Fig. 6*B*) and protein (Fig. 6*C*) into the bronchoalveolar lavage were determined. Naphthalene-exposed mice exhibited weight loss and increased bronchoalveolar lavage white blood cell and protein concentrations. The lungs were harvested for histological (Fig. 6*D*) and immunolabeling (Fig. 6*E*) analysis. Using the club cell marker CC10, we established that naphthalene exposure induced near total ablation of club cells of the proximal and distal conducting airways within 5 days. At *day 5*, the epithelium had begun to reestablish back to a mucociliary monolayer; by *day 14*, the epithelia had recovered from the initial naphthalene injury. This time course is consistent with current literature regarding this model (73). Similarly, immunolabeling analysis of the pan-epithelial marker E-cadherin showed an initial loss of epithelial cells on *day 2* followed by complete reepithelialization on *day 14*.

**Figure 7.**
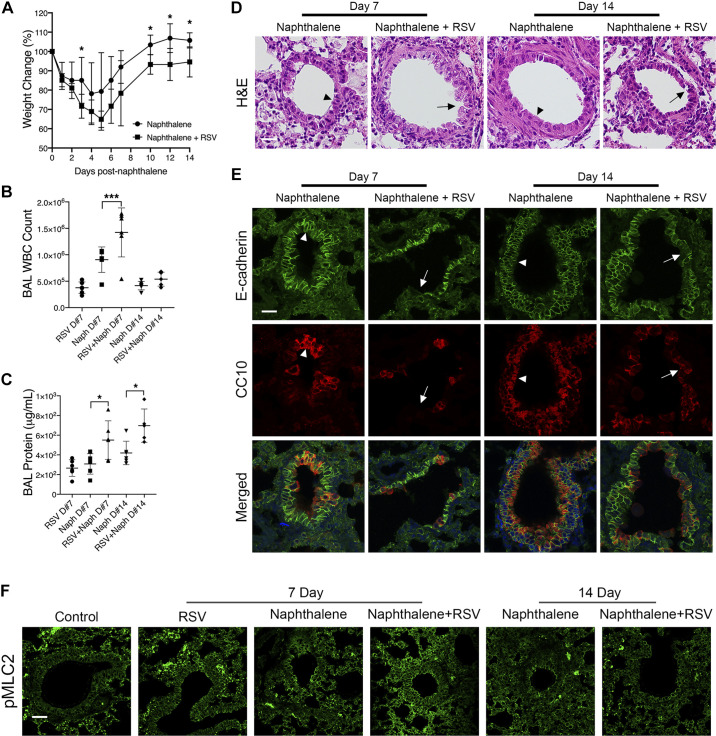
Respiratory syncytial virus (RSV) infection delays airway epithelial wound healing in vivo. Mice were intraperitoneally administered control PBS or naphthalene on *day 0*. On *day 2*, select mice received an intranasal inoculation of RSV. Mice were weighed daily, and the percentage of body weight change was calculated from body weights at *day 0* (*A*). Mice were euthanized on *days 7* and *14* after naphthalene injection (*days 5* and *12* after RSV infection). Transmigrated leukocytes [white blood cells (WBC)] were counted in the bronchoalveolar lavage (BAL; *B*). Total protein concentration was determined in collected BAL samples (*C*). Photomicrographs of hematoxylin and eosin (H&E)-stained lung tissue section were examined (*D*). Immunohistochemical analyses of epithelial and club cell markers (*E*) and phospho-myosin light chain 2 (pMLC2; *F*) were conducted. Arrows in *D* and *E* indicate histopathological changes including peribronchial inflammation and epithelial thickening. Arrowheads show areas of denuded epithelium that recovered by *day 14*. Scale bar = 40 μm. Data are presented as means ± SD; *n *=* *10 mice/group. ANOVA was conducted. *P < 0.05; ***P < 0.001.

To recapitulate our findings that RSV attenuates wound healing in an in vivo system, C57BL/6 mice were intraperitoneally administered a single dose of naphthalene (or corn oil vehicle). Two days later, some of the animals received an intranasal inoculation of 9.6 × 10^6^ plaque-forming units of rrRSV. Mice were euthanized on *days 7* and *14* after naphthalene exposure (corresponding to *days 5* and *12* of RSV infection). Animals exposed to both naphthalene and RSV presented with more severe weight loss (Fig. 7*A*) and increases in leukocyte numbers (Fig. 7*B*) and protein infiltration into the bronchoalveolar lavage (Fig. 7*C*) compared with naphthalene only-exposed animals. Hematoxylin and eosin staining of paraffin-embedded and sectioned lungs revealed a delay in epithelial healing as evidenced by sustained loss of club cells and epithelial “breaks” that had resolved in naphthalene only-exposed lungs by *day 14* (Fig. 7*D*). Immunolabeling analysis of the pan-epithelial marker E-cadherin and club cell marker CC10 revealed that RSV delays reepithelialization after naphthalene injury (Fig. 7*E*).

To investigate the role of the RhoA pathway in our in vivo system, we conducted immunohistochemical analysis of paraffin-embedded lungs with pMLC2, as the downstream signaling marker of RhoA (Fig. 7*F*). At *day 7*, there were increased concentrations of pMLC2 in naphthalene + RSV animals compared with naphthalene-only or RSV-only mice. All groups had diminished levels at *day 14*. This is congruent with our in vitro findings that showed an increase in pMLC2 in RSV-infected cells.

## DISCUSSION

**Figure 8.**
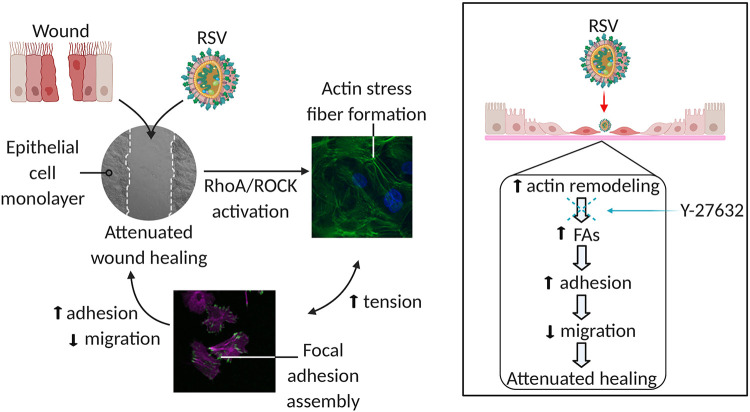
Schematic representation of involved pathways. Respiratory syncytial virus (RSV) infection attenuates wound healing following epithelial cell injury by inducing actin cytoskeletal remodeling. RSV infection activates small GTPase RhoA, which promotes actin stress fiber formation. At the same time, increased focal adhesion (FA) development occurs due to increased cellular tensions caused by RSV. These changes cause cells to anchor to the extracellular matrix, which hinders migration and slows wound healing. Y-27632, a Rho-associated kinase (ROCK) inhibitor, reduces stress fiber formation and FA protrusions, which allows for typical cell migration and wound healing. (Figure was created with BioRender.com.)

In this study, we found that RSV infection significantly delays wound healing in immortalized and primary human airway epithelial cells in vitro. This was associated with increased cell adhesion to the ECM and marked reorganization of the actin cytoskeletal architecture in infected cells. In cells exposed to RSV, there was a striking increase in the mean length of FAs (Fig. 8).

The ability of the epithelium to repair itself after injury is essential for resolution of airway disease. Prompt healing of the airway epithelium is crucial to prevent infiltration of inhaled allergens and pathogens into the subepithelial space and to minimize inflammatory responses in the lungs (11, 12). In a noninfected cell, F-actin allows for cell migration through polymerization and depolymerization of filaments (74), a process that is essential for the development of stress fibers and FAs. Stress fibers are bundles of F-actin and other cytoskeletal proteins that are anchored at one or both ends by FAs, a group of proteins that also connects to the ECM. In a noninfected cell, FAs push the plasma membrane, and stress fibers pull the cell body in order to promote migration of the cell (75). It is this repetitive cycle of forward extension and attachment followed by cell contraction and rear release that allows for cell migration (71, 76). When a cell is infected or under forces, new components and proteins are recruited to the FAs in order to secure cells to the ECM. Furthermore, FA proteins expose modified sites to facilitate new interactions with the ECM (77). Cell-matrix interactions must be tightly monitored and controlled in order to allow cells to move. Our study showed that RSV infection induces the formation of prominent stress fibers, accompanied by assembly of large FA plaques (Fig. 3). Overly tense FAs, as seen with RSV infection, can prevent rear retraction (71). In RSV infection, the actin cytoskeleton dysfunction and increased stress fiber and FA formations could cause the cells to anchor to the ECM, which hinders migration. Similar results in which retention of actin stress fibers in the presence of increased cortical actin was correlated with elevated cellular stiffness has been shown in rubella virus (78).

A fine balance between actin polymerization and adhesion is needed for proper migration of epithelial cells for wound repair mechanisms. Actin turnover is tightly maintained by a family of actin regulators, small GTPases (38–40, 79). RSV is known to activate small GTPase RhoA, a master regulator of actin stress fiber formation (80). Studies have implicated excessive RhoA activation with slowed migration of epithelial cells (81, 82), astroglioma cells (83), squamous cell carcinomas (84), and fibroblasts (85, 86). The role of RhoA was probed directly, through downstream effectors, and by inhibition of its effector, ROCK. ROCK has been identified as a therapeutic target in the treatment of asthma and chronic obstructive pulmonary disorder (87).

Our study showed that RhoA and pMLC2 expression increased during RSV infection (Figs. 3 and 4). This phenomenon has also been described during rotavirus infection in the intestinal epithelium, albeit at an earlier time point after infection (88). In addition, there was increased pMLC2 localization to the cell membrane following RSV. Previous research in vascular endothelial cells showed that actin bundle formation occurs in the cell periphery, and, later, stress fiber formation occurs in the perinuclear cytoplasm (89). Therefore, perhaps this change in localization following RSV infection is to preemptively allow for stress fiber formation. Furthermore, actin filament formation following activation of RhoA was attenuated by incubation with the small-molecule ROCK inhibitor Y-27632 (Fig. 5). Consequentially, hyperadhesiveness mediated by the assembly of FA plaques was abrogated, and cell migration was restored. Similarly, previous studies have shown that Y-27632 suppresses airway hyperresponsiveness induced by RSV infection (90), without altering viral replication (91).

RSV does not induce cell necrosis or apoptosis, as indicated by lactate dehydrogenase release and caspase-3 cleavage assays, as previously described (33). During wound healing, epithelial cells proliferate to increase the number of cells available to cover the wound and, afterward, differentiate into mature epithelial cells. We also did not observe RSV-induced inhibition of proliferation. This, however, conflicts with some studies that have suggested that RSV inhibits proliferation (92). However, studies have also shown that persistence of RSV may promote proliferation (93). Available data regarding the effects of RSV on cell migration are limited and conflicting, and the underlying mechanisms remain unknown. The few existing in vitro studies have reported contradictory data by showing either inhibited migration of RSV-infected hepatocellular carcinoma cells (94) or accelerated motility of RSV-exposed lung adenocarcinoma cells (95). Choi et al. performed a Transwell migration assay in which cells had to squeeze through pores rather than solely cross a surface, which may account for the differences seen in hepatocellular carcinoma cells. Mehedi et al. used A549 cells, which perhaps may explain the discrepancy in lung adenocarcinoma cells.

The present study, while describing an essential mechanism of airway epithelial restitution, has limitations. The naphthalene-induced airway injury used in this study results in specific ablation of club cells of the airway only and may not fully recapitulate an epithelial lesion. However, previous studies have demonstrated that club cells account for 15% of proliferating airway epithelial cells in the terminal bronchioles and, thus, are vital for maintaining the distal airways after injury (69, 96). Therefore, we consider naphthalene a suitable model for studying airway injury in a murine model. Another limitation of this study lies in the possibility that multiple small GTPases may contribute to RSV-dependent inhibition of epithelial cell migration. Although RhoA is primarily responsible for the assembly of actin cables, it is possible that in parallel, upstream regulators of actin polymerization such as Rac1 and Cdc42 may also play roles in attenuating cell migration (97, 98). However, given numerous RhoA activation studies and the effect of pMLC2 activation and ROCK inhibition on migration, it is apparent that these other regulators do not play as large of a role. This is an exciting avenue for investigation in future studies. Finally, in our in vitro models, RSV infection had to occur before wound scratching, as 16HBE and primary cells need 48 h to become infected and wound repair would have been completed before infection. Epithelial injury before RSV infection is more representative in vivo, which was done in the mouse experiments. Notably, both our in vitro and in vivo experiments suggest that RSV-induced attenuation of cell migration occurs through RhoA-ROCK-MLC pathway activation leading to actin cytoskeletal remodeling.

In conclusion, we demonstrate how a common viral pathogen alters cytoskeletal derangement through stress fiber and FA formation and consequentially impairs the migration of epithelial cells necessary for healing upon injury. This study elucidates a novel role of RSV as a potent inhibitor of airway epithelial cell motility by mechanisms involving remodeling of the actin cytoskeleton and enhancement of cell-ECM adhesion. Understanding the mechanisms by which RSV affects airway epithelial cell migration will deepen our understanding of the ramifications of RSV infection and will lay the ground for future studies such as the role of viral infection in ventilator-induced lung injury and environmental exposures as well as the long-term consequences of infection. Proteins that maintain cell membrane integrity, mobility, and adhesion are of particular importance in RSV-induced airway injury, and the insights gained here may help design future translational approaches to identify potential targets for therapeutic intervention to treat acute and chronic sequelae of RSV infection.

## SUPPLEMENTAL DATA

Supplemental Fig. S1: https://doi.org/10.6084/m9.figshare.14579871.v1.

## GRANTS

This work was supported by the Mark Lauer Pediatric Research Grant, Cleveland Clinic Children’s (to F. Rezaee), as well as by National Institutes of Health (NIH) Grants K08AI112781 and R01HL148057 (to F. Rezaee) and R01061007 (to G. Piedimonte). This work used the Leica SP8 confocal microscope that was purchased with funding from NIH SIG Grant S10OD019972.

## DISCLAIMERS

The authors have no financial relationship with a biotechnology and/or pharmaceutical manufacturer that has an interest in the subject matter or materials discussed in the submitted manuscript.

## DISCLOSURES

No conflicts of interest, financial or otherwise, are declared by the authors.

## AUTHOR CONTRIBUTIONS

F.R. conceived and designed research; D.T.L., N.G., A.R., T.J.H., and F.R. performed experiments; D.T.L., N.G., A.R., T.J.H., G.P., and F.R. analyzed data; D.T.L., N.G., G.P., and F.R. interpreted results of experiments; N.G. and F.R. prepared figures; F.R. drafted manuscript; D.T.L. and F.R. edited and revised manuscript; D.T.L., N.G., A.R., T.J.H., G.P., and F.R. approved final version of manuscript.
